# Allorecognition of HLA-C Mismatches by CD8^+^ T Cells in Hematopoietic Stem Cell Transplantation Is a Complex Interplay between Mismatched Peptide-Binding Region Residues, HLA-C Expression, and HLA-DPB1 Disparities

**DOI:** 10.3389/fimmu.2016.00584

**Published:** 2016-12-12

**Authors:** Florence Bettens, Stéphane Buhler, Jean-Marie Tiercy

**Affiliations:** ^1^National Reference Laboratory for Histocompatibility, Department of Genetic and Laboratory Medicine, University Hospitals Geneva, Geneva, Switzerland

**Keywords:** HLA-C, alloreactivity, cytotoxic T lymphocytes, hematopoietic stem cell transplantation, mixed lymphocyte reaction

## Abstract

HLA-C locus mismatches (MMs) are the most frequent class I disparities in unrelated hematopoietic stem cell transplantation (HSCT) and have a detrimental impact on clinical outcome. Recently, a few retrospective clinical studies have reported some variability in the immunogenicity of HLA-C incompatibilities. To get better insight into presumably permissive HLA-C MMs, we have developed a one-way *in vitro* mixed lymphocyte reaction (MLR) assay allowing to quantify activated CD56^−^CD137^+^CD8^+^ lymphocytes in HLA-C incompatible combinations. T cell-mediated alloresponses were correlated with genetic markers such as HLA-C mRNA expression and the number of amino acid (aa) MMs in the α1/α2 domains (peptide-binding region). Because of the high rate of HLA-DPB1 incompatibilities in HLA-A-, B-, C-, DRB1-, and DQB1-matched unrelated HSCT patient/donor pairs, the impact of HLA-DPB1 mismatching, a potential bystander of CD4^+^ T cell activation, was also considered. Heterogeneous alloresponses were measured in 63 HLA-C-mismatched pairs with a positive assay in 52% of the combinations (2.3–18.6% activated CTLs), representing 24 different HLA-A~B~DRB1~DQB1 haplotypes. There was no correlation between measured alloresponses and mRNA expression of the mismatched HLA-C alleles. The HLA-C*03:03/03:04 MM did not induce any positive alloresponse in five MLRs. We also identified HLA-C*02:02 and HLA-C*06:02 as mismatched alleles with lower immunogenicity, and HLA-C*14:02 as a more immunogenic MM. A difference of at least 10 aa residues known to impact peptide/T cell receptor (TCR) binding and a bystander HLA-DPB1 incompatibility had a significant impact on CTL alloreactivity (*p* = 0.021). The same HLA-C MM, when recognized by two different responders with the same HLA haplotypes, was recognized differently, emphasizing the role of the T-cell repertoire of responding cells. In conclusion, mismatched HLA-C alleles differing by 10 or more aas in the peptide/TCR-binding region, when occurring together with HLA-DPB1 incompatibilities, should be considered as high-risk MMs in unrelated HSCT.

## Introduction

HLA class I molecules are expressed on almost all nucleated cells and play a key role in the immune responses to pathogens, cancer cells, and autoantigens. In addition to their extremely high level of allelic polymorphism, they are also characterized by variable levels of expression potentially influencing their function. Although HLA-C antigens are expressed at a lower level compared to HLA-A and B antigens (1–4), they are also well recognized by alloreactive T cells and thus are *bona fide* transplantation antigens. Several studies have recently reported variability in the expression of different HLA-C serotypes, as determined at the cell surface or mRNA steady-state levels (5–8). Variability within HLA-A serotypes has also been recently reported (9). A search for genetic markers of HLA-C expression led to the description of two relevant polymorphisms. First, a SNP located 35 kb (*rs9264942*) upstream of the HLA-C locus has been reported to correlate with the level of HLA-C expression and with the control of HIV viremia (7). HLA-C allotypes with a higher expression marked by the −35C genotype have been shown to correlate with a better control of HIV infection and to a more efficient recognition by CTLs (5, 7). The second polymorphism in the 3′-UTR of HLA-C gene is the 263del/ins variant affecting the miRNA-148a binding and controlling at least partly HLA-C mRNA stability (8). Control of HLA-C expression is most likely more complex because of the lack of consensus between genetic markers and expression levels reported by different studies. For example, the C*14:02 allotype, reportedly classified as high if not the highest (5) expression allele, is characterized by the 263ins variant associated with low expression (8). The correlation of these genetic markers with expression levels has been challenged by a few other studies (6, 10, 11). The studies analyzing HLA-C cell surface expression or mRNA steady state were hampered by the fact that it was not possible to discriminate between the two HLA-C alleles in heterozygous donors. By using group-specific polymerase chain reaction (PCR), individuals expressing the same HLA-C allele did show variability in mRNA steady-state amounts within a given serotype that could possibly be correlated with specific haplotypes (6).

As determined in retrospective clinical studies in mismatched unrelated hematopoietic stem cell transplantation (HSCT), immunogenicity of HLA-C mismatches (MMs) has been reported to be affected by specific amino acid (aa) residues in the peptide-binding region (PBR). In particular, HLA-C MM involving residue 116 has been reported to be associated with higher risk of aGVHD and mortality (12–14).

Two studies, so far, have tested the impact of HLA-C expression level on the clinical outcome of 9/10-matched unrelated HSCT. As a proxy of HLA-C expression, both studies used the previously reported mean fluorescence intensity (MFI) values of anti-HLA-C DT9 antibody binding determined in 200 African-American and in 50 European-American – mostly heterozygous – donors (5). Using 1,975 9/10-matched HSCT of the International Histocompatibility Working Group in HCT, Petersdorf et al. (15) compared patients with low expression mismatched C*03 and C*07 allotypes and high expression C*01 and C*14 allotypes and found that high expression mismatched HLA-C alleles were associated with increased mortality. On the other hand, analyzing 1,965 pairs with single HLA-C MMs, Morishima et al. (16) did not find any correlation between increasing level of expression of patient’s mismatched HLA-C allele and aGVHD or TRM risk, although patients’ HLA-C*14:02 was significantly associated with poor outcome. The same MFI values have also been applied to analyze a possible correlation with *in vitro* CTLp frequency to single HLA-C MMs. CTLp outcome was reported to correlate both with the expression of the donor and of the recipient mismatched HLA-C antigens (17).

Besides HLA-C MMs, HLA-DPB1 MMs are the most frequent disparities in unrelated HSCT with detrimental effects on acute GVHD and other transplantation-related risks (18–20). Specific HLA-DPB1 MMs with higher potential for T cell allorecognition, as well as expression levels of HLA-DPB1 variants, have been reported to be associated with acute GVHD in unrelated HSCT (21, 22).

In this study, we investigated the CTL alloresponse induced by single HLA-C MMs by quantifying CD8^+^CD56^−^-activated T lymphocytes in an *in vitro* mixed lymphocyte reaction (MLR) assay. Our aim was to correlate the immunogenicity of HLA-C MM with HLA-C mRNA expression levels, with disparities in aas known to be relevant for peptide binding and/or T cell receptor (TCR) recognition, and also with concomitant HLA-DPB1 incompatibilities.

## Materials and Methods

### Patients and Donors

Peripheral blood mononuclear cells (PBMCs) were purified using standard Ficoll procedure from blood collected from patients and donors who have been analyzed by the National Reference Laboratory for Histocompatibility (LNRH) for unrelated HSC donor searches. HLA high resolution typing was performed by reverse PCR-sequence-specific oligonucleotides typing on microbeads arrays (One Lambda, Canoga Park, CA, USA), by PCR-sequence-specific primers (Genovision, Milan Analytika AG, Switzerland), and by monoallelic sequencing (Protrans, Hockenheim, Germany). This study has been approved by the ethical committee of the Geneva University Hospitals (reference #08-208R). For many years, *in vitro* cellular assays (CTLp and MLR) have been performed at LNRH as part of the routine histocompatibility testing for unrelated HSC donor searches.

### Mixed Lymphocytes Reactions

For allogenic stimulation, one-way MLRs were performed using thawed cryopreserved PBMC, which were cryopreserved in RPMI 1640 medium (Gibco Life Technologies, Oslo, Norway) supplemented with l-glutamine, penicillin and streptomycin (Gibco), 10% dimethylsulfoxid (Merck, Darmstadt, Germany), and 20% fetal calf serum (Gibco). Responder cells (2 × 10^6^) were stimulated at a ratio of 1:1 with 30 Gy irradiated stimulator cells in RPMI 1640 medium (Gibco) supplemented with l-glutamine, penicillin and streptomycin (Gibco), and 10% human AB serum (own preparation). Twenty units per milliliter rIL-2 (Peprotech, London, UK) were added at days 3, 7, and 11. After 13 days of culture, responding T cells were restimulated overnight with irradiated PKH-2 (Sigma-Aldrich, Buchs, Switzerland)-labeled PHA blasts obtained by activation of non-irradiated stimulatory PBMCs with 1 μg/ml PHA (Gibco). The percentage of CD137^+^PKH-2^−^CD8^+^CD56^−^ viable T cells was quantified by flow cytometry using APC-labeled anti-human CD8a, PerCP/Cy5.5 anti-human CD56 (BioLegend, Fell, Germany), and FITC-labeled anti-human CD137 (Milteny Biotec, Bergisch-Gladbach, Germany) antibodies, as well as APC- and FITC-labeled murine IgG1 isotype controls (BD Bioscience, Allschwil, Switzerland). As controls, half of the cultures were also restimulated with autologous PHA blasts. The percentage of activated CD8^+^ T cells referred as Δ%CD137^+^CD8^+^ equals the percentage of CD137^+^CD8^+^CD56^−^ cells measured in cultures restimulated with stimulator cells minus the percentage of CD137^+^CD8^+^CD56^−^ cells measured in cultures restimulated with autologous cells (23). Data acquisition was performed on 5,000 gated CD8^+^CD56^−^ cells using the ACCURI-C6 cytometer (BD) and the CFLOWPLUS analysis software.

An MLR assay with Δ%CD137^+^CD8^+^ ≥2% was considered as positive, this threshold corresponding to the variability observed in unstimulated cultures. As positive controls to monitor responsiveness of the responder cells and ability of stimulator cells to induce an alloresponse, responder cells were stimulated in parallel cultures with allogeneic stimulatory cells mismatched for several HLA-A, B, and C alleles, and similarly, stimulator cells were used to stimulate allogeneic HLA-A-, B-, and C-mismatched responder cells. In this study, only MLRs with a positive allogenous control were considered. Of the 63 MLRs, 12 could be repeated at different dates, as a way to control for reproducibility of our experiments, because enough cells had been cryopreserved. With two exceptions, all repeated cultures (eight duplicates and two triplicates) allowed the same positive versus negative alloresponse discrimination (SD of measured alloreactivity varying between 0.11 and 4.66, including seven replicates with SD below 1.8).

### HLA-C mRNA Quantification

Total RNA was extracted from thawed stimulator cells prior to irradiation using the RNeasy Micro kit (Qiagen, Valencia, CA, USA) and according to the manufacturer’s instructions. RNA was reverse transcribed with the M-MLV H-Minus (Promega, Madison, WI, USA) reverse transcriptase and random oligo dT 15mer-primers in the presence of rRNAsin (Promega). HLA-C cDNA was quantified relative to 18S RNA, as previously described (6), using SYBR Green/Rox (Abgene, Epsom, UK)-based quantitative real-time PCR on a real-time PCR System 7300 using the SDS software version 1.3.10.2 (Applied Biosystems). The results were expressed as 100/ΔCt (where ΔCt is Ct HLA-C − Ct 18S).

### Differences in PBR Residues and Statistical Analyses

HLA-C mature protein sequences were retrieved from the IMGT/HLA database (http://www.ebi.ac.uk/ipd/imgt/hla/) and used to compute the difference in aa for each allele pair (i.e., the number of mismatched residues in the PBR between the stimulator and the responder). Each residue was categorized as forming, or not, the pocket-like structures of the PBR, as being involved, or not, in peptide binding according to various biological, chemical, or physical properties [see Ref. (24) and references therein] and as being a putative recognition site for the TCR (25).

Logistic and linear modeling analyses were performed using the R statistical computing environment to assess individually or jointly the association(s) between the number of mismatched residues, HLA-DPB1 matching, expression of the stimulator allele and variation at positions 80 and 116 (i.e., the predictor or independent variables), and alloreactivity [i.e., the response or dependent variable considered, respectively, as (a) positive or negative or (b) measured along a continuous axis]. The second approach allowed avoiding too strong assumptions based on an arbitrary cutoff value of alloreactivity. The linear models were validated, whenever appropriate, using diagnostic plots.

## Results

### Heterogeneity of the CTL Alloresponse to HLA-C Alleles

The CTL alloresponse was measured in 63 *in vitro* MLR assays including 18 different target HLA-C alleles. With two exceptions, all HLA-C MMs differed in the α1/α2 domains. The 18 target alleles included all frequent C allotypes present in populations of European origin except C*08:02. Seven MLR combinations consisted in HLA-C *allele* MMs within the C*02 (two pairs) and C*03 serotype (five pairs). The C*02 combinations consisted of the C*02:02/02:29 incompatibility that differs at residue 265 in the α3 domain. The C*03 combinations were C*03:03/03:04 MMs differing by a single residue at position 91 in the α1 domain. The remaining 56 pairs consisted in HLA-C *antigen* MMs (26).

**Figure 1:**
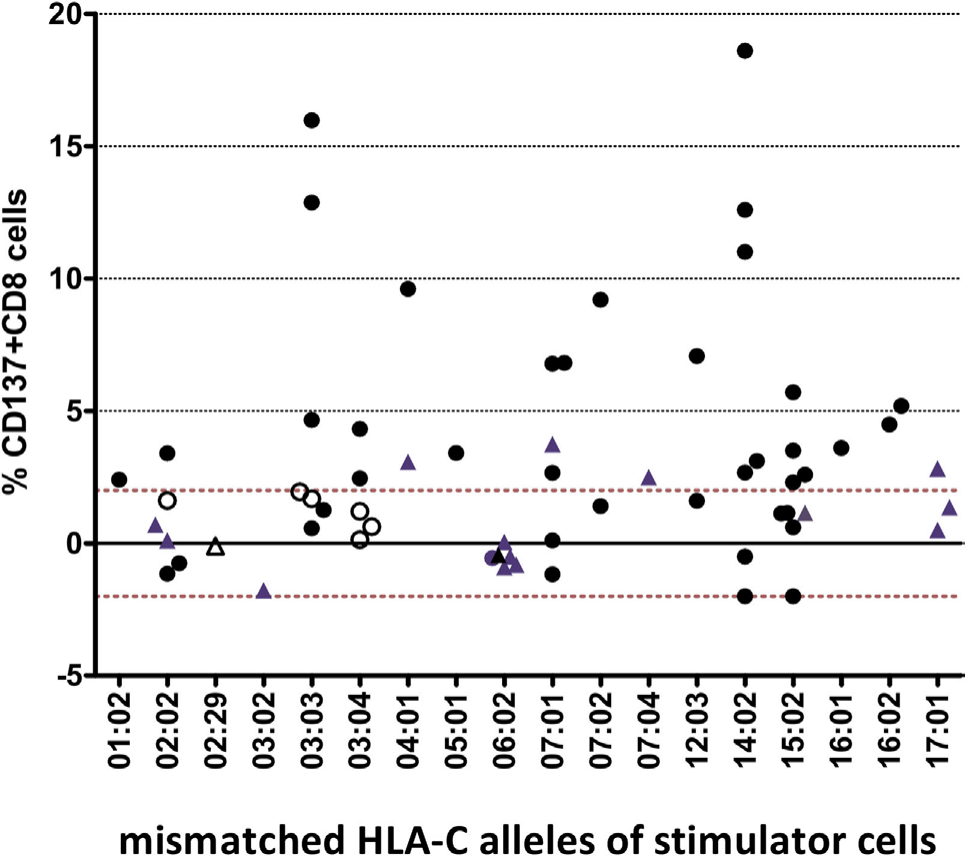
**Heterogenous alloresponse tested by *in vitro* mixed lymphocyte reaction (MLR)/flow cytometry assays in 63 responder/stimulator cell combinations that were characterized by a single HLA-C mismatch**. The mismatched HLA-C allele of the stimulator cells and the %ΔCD137^+^CD8^+^ cells induced after restimulation at day 14 are indicated (see Materials and Methods). The cutoff of 2% ΔCD137^+^CD8^+^ cells is indicated by the dashed line. Activation of CD8^+^ NK cells (ranging between 0.2 and 1.2% CD8^+^CD56^+^CD137^+^ cells, results not shown) was not taken into consideration. Overall, 24 different shared HLA-A~B~DRB1~DQB1 haplotypes were tested (data not shown). Twelve MLRs were repeated but dotted as single mean %ΔCD137^+^CD8^+^ values. Black dots represent MLRs with HLA-C MMs located in α1/α2 domains and HLA-DPB1 MMs. Blue triangles represent MLRs with HLA-C MMs located in α1/α2 domains and matched HLA-DPB1. Open circles represent MLRs with C*03:03/03:04 MMs and C*03:04/03:03 MMs, all HLA-DPB1 MMs. Open triangles represent MLRs with HLA-C MMs located outside α1/α2 domains (C*02:29/02:02, C*02:02/02:29) and matched HLA-DPB1.

For each of the 18 different HLA-C target alloantigens, 1–9 MLRs were performed with a total of 24 different HLA-A~B~DRB1~DQB1 shared haplotypes. The overall results (Figure 1) showed a heterogeneous response toward single HLA-C MM: 52% of all combinations led to a positive assay within a range of 2.3–18.6% CD137^+^CD8^+^ T cells, whereas 48% were negative (<2%).

The CTL alloresponses toward the same HLA-C MM show that the C*14:02 target alloantigen is recognized at a higher level, with five of seven pairs leading to a positive response varying between 2.7 and 18.6%. Similarly, the C*03:03 target alloantigen was well recognized by non-C*03:04 responders (3/5 positive tests, 4.7–15.9%). Furthermore, 6/6 C*06:02 and 5/6 C*02:02 MMs were not able to induce a CD8^+^ T cell response. Only one C*02:02 target alloantigen was weakly recognized by 3.4% CD137^+^CD8^+^ T cells. All of the five C*03:03/03:04 MM pairs were “silent” MMs, either with the C*03:03 (two pairs) or the C*03:04 (three pairs) alleles as target alloantigens (open circles in Figure 1).

### Impact of HLA-C Expression

**Figure 2:**
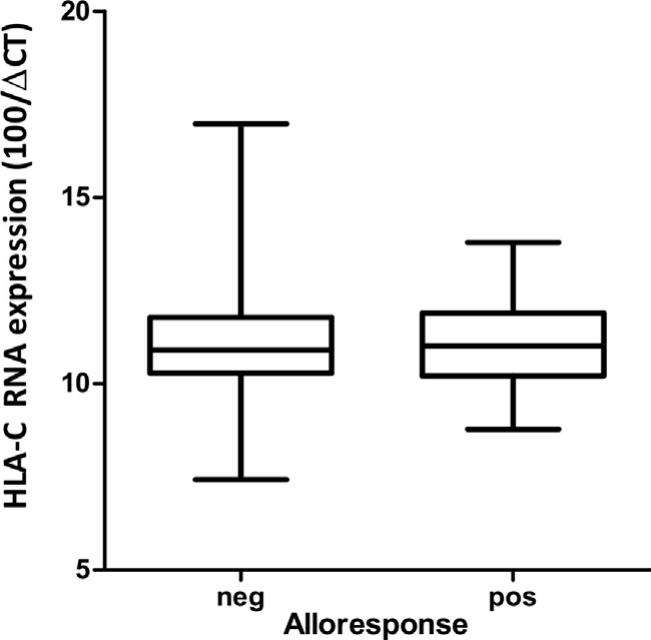
**Box and whisker plots of HLA-C mRNA expression quantified by RT-PCR in stimulator cells at day 0 inducing a negative versus a positive (cutoff = 2% ΔCD137^+^CD8^+^ cells) alloresponse**.

**Figure 3:**
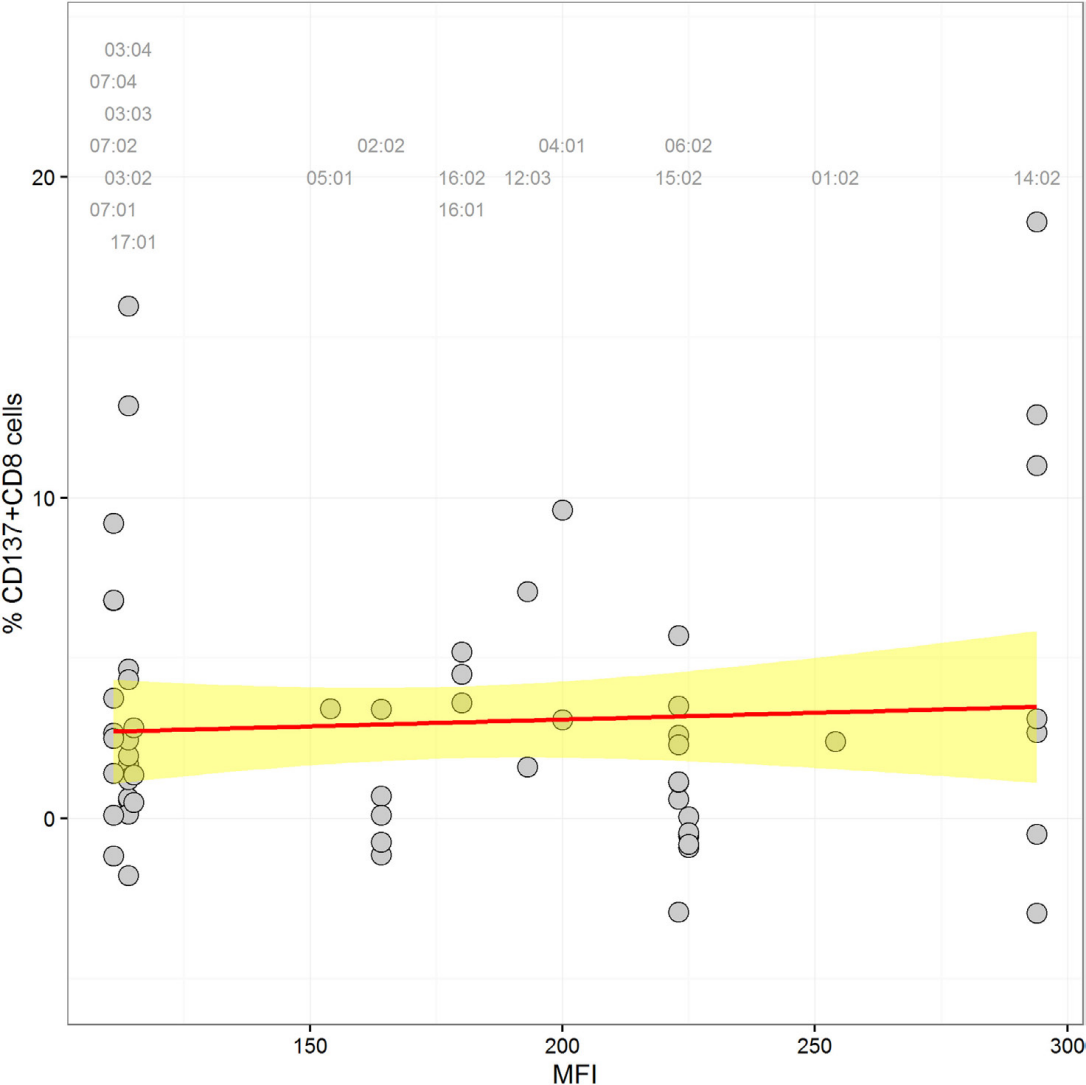
**Scatter plot of HLA-C expression [mean fluorescence intensity (MFI) values according to Ref. (5, 15)] and alloreactivity (%ΔCD137^+^CD8^+^ cells)**. A label is plotted for each stimulator allele at the top of the graph, indicating its corresponding MFI value. The linear regression through the data points is shown in red, and the confidence interval is in yellow (coefficient = 0.004, *p* = 0.637).

In order to detect a possible correlation between CTL alloresponses and levels of HLA-C expression, we quantified mRNA steady-state levels of the stimulator PBMCs at day 0 of the MLR. As presented in Figure 2, no correlation between the CTL alloresponse and quantified HLA-C mRNA steady-state levels could be disclosed when data were analyzed altogether (non-significant coefficients in single and multiple linear regressions, results not shown). Exclusion of the 02:29/02:02, 02:02/02:29, 03:04/03:03, and 03:03/03:04 pairs (single MM in α3 domain or in α2 domain but not seen by the TCR) did not significantly alter the results. We also classified the 63 assays performed in our study by using previously published quantitative measurement of MFIs (5, 15) to impute expression levels of HLA-C allotypes. The results do not show any significant correlation between the imputed expression level of the target HLA-C antigen and the CTL alloresponse (*p* = 0.637, Figure 3).

### Impact of Mismatched aa Residues Affecting Peptide/TCR Binding

**Figure 4:**
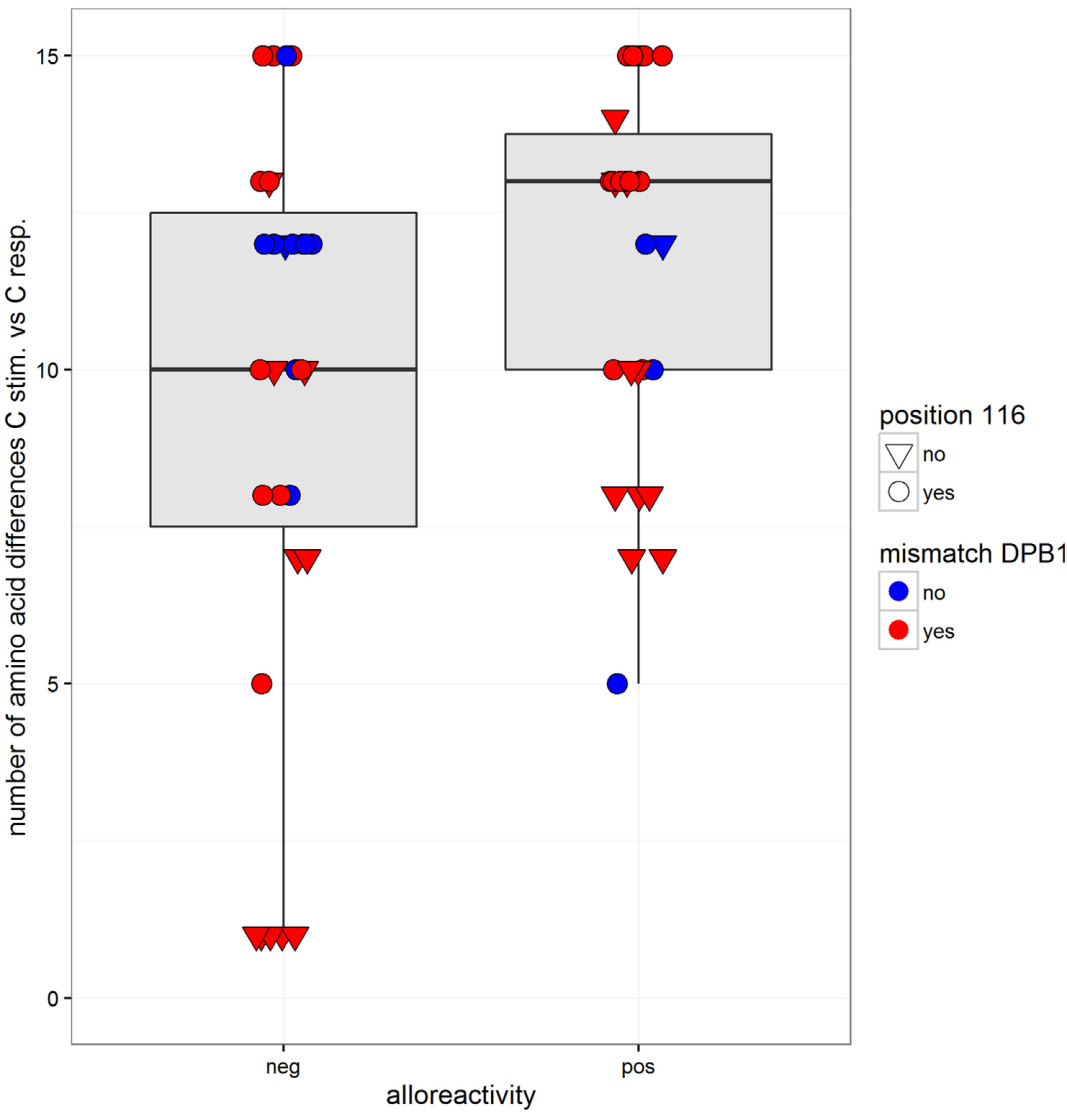
**Box and whisker plots of the number of mismatched residues at HLA-C between the stimulator and the responder alleles and alloreactivity defined as negative or positive (cutoff = 2% ΔCD137^+^CD8^+^ cells)**. The boxes correspond to the interquartile range, the median is the thick line inside the box, and whiskers extend up to observations that are outside the box for less than 1.5 times the interquartile range. No outliers to these limits were observed. The observations are also plotted individually with information on two predictor variables: matching at HLA-DPB1 is indicated by two different colors (blue for matched and red for mismatched HLA-DPB1), while variation at position 116 is indicated by the shape of the dots (a reversed triangle for matched and a circle for mismatched).

**Figure 5:**
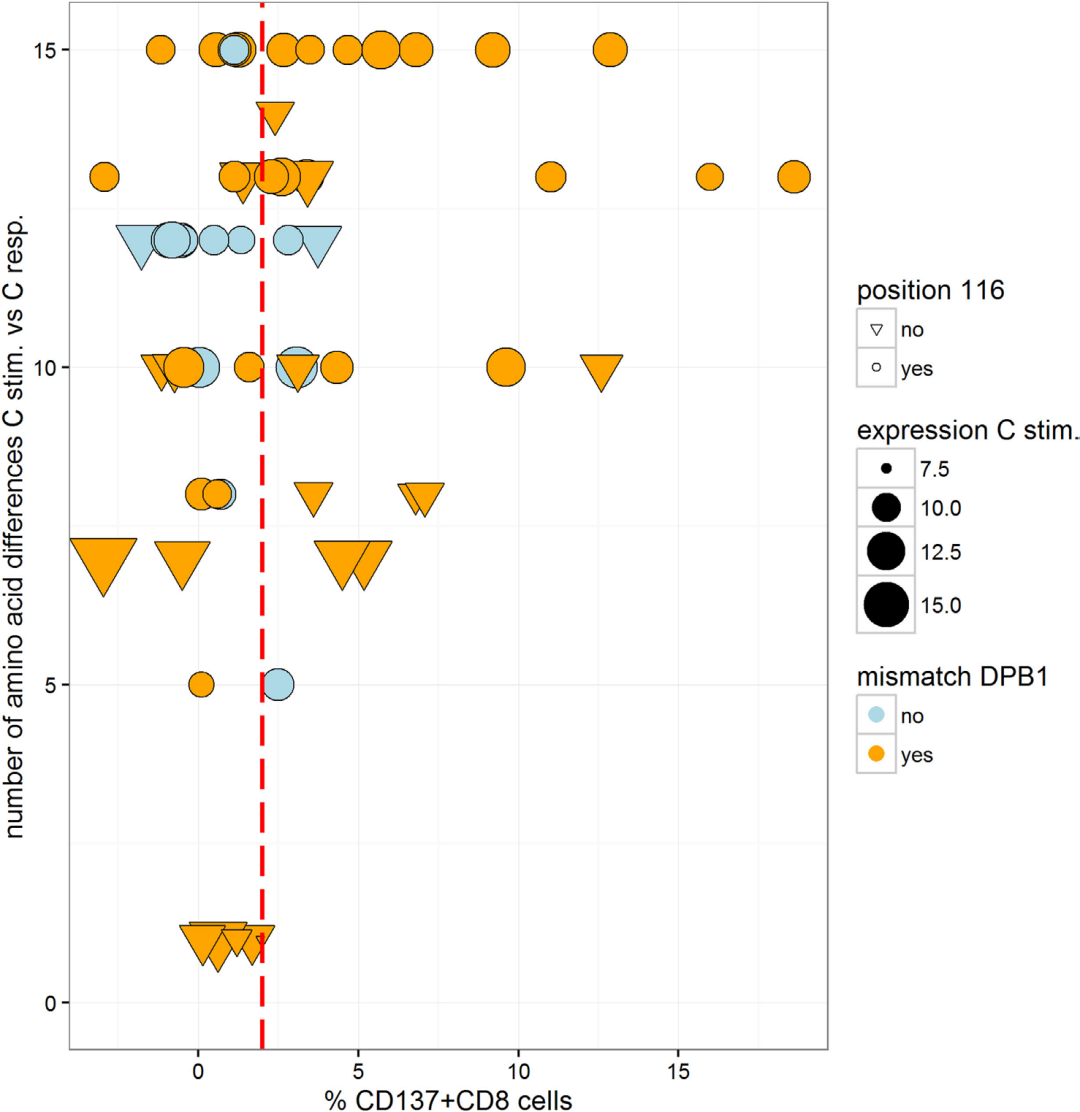
**Scatter plot of the number of mismatched residues at HLA-C between the stimulator and the responder allele and alloreactivity (%ΔCD137^+^CD8^+^ cells)**. Information on several predictor variables is plotted: matching at HLA-DPB1 is indicated by two different colors (blue for matched and orange for mismatched HLA-DPB1), mRNA expression of the stimulator HLA-C allele is indicated by the size of the dots, while variation at position 116 is indicated by the shape of the dots (a reversed triangle for matched and a circle for mismatched). The cutoff value considered for positive/negative alloreactivity is shown by the red dotted line.

In order to analyze the impact of functionally relevant aa MMs, we compared the alloresponse with the number of aa differences in the α1/α2 domains. The two pairs with a C*02:02/02:29 MM were excluded because the two alleles have identical sequences in the α1/α2 domains. As determined in 61 pairs, between 1 and 15 aa differences were recorded (mean = 10.6, median = 12, not shown). On average, the number of mismatched residues was higher in the MLR pairs that resulted in a positive CTL alloresponse (Figure 4 and logistic regression, coefficient = 0.158, *p* = 0.037). The mismatched residues affected predominantly those involved in peptide binding relative to the TCR contact residues. Taking into account both the number of aa differences (coefficient = 0.169, *p* = 0.026) and the HLA-DPB1 matching (coefficient = 1.27, *p* = 0.06) as predictor variables, only the aa differences appeared to have an impact (additive model, interaction not significant). However, when the C*03:03/03:04 MM pairs were omitted, the aa differences were not significant anymore, while HLA-DPB1 became borderline significant (coefficient = 1.44, *p* = 0.048). Measuring alloreactivity along a continuous axis and testing several predictor variables (Figure 5), only HLA-DPB1 matching was significant either considering the 61 pairs (linear regression, coefficient = 2.82, *p* = 0.031) or omitting C*03:03/03:04 MM pairs (coefficient = 2.74, *p* = 0.039).

The ratio of pairs with aa116 MMs was similar in negative (20/31) and positive MLRs (19/30). Among aa116 MM pairs, those with HLA-C expression above the mean value (11.23) had a higher rate of positive MLR (53% compared to 31% in pairs with low expression, not significant). MMs at positions 77 and 80 were not associated with T cell alloreactivity (data not shown).

Of 44 pairs with ≥10 HLA-C aa MMs, 24 induced a positive alloresponse and 20 were negative. A strong correlation was disclosed with the HLA-DPB1-matching status since 87.5% of the positive pairs and 55% of the negative pairs were HLA-DPB1 incompatible (Figure 4). Altogether, positive CTL alloresponses occurred more frequently in HLA-C-mismatched combinations that differed by ≥10 residues and were HLA-DPB1 incompatible (*p* = 0.021, Fisher’s test). It is relevant to note that, as shown in Figure 1, none of the HLA-DPB1-matched pairs (blue triangles) induced a high alloreactivity, with all HLA-DPB1-matched pairs showing <3.75% CD137^+^CD8^+^ T cells.

### Role of Responder Cells

**Figure 6:**
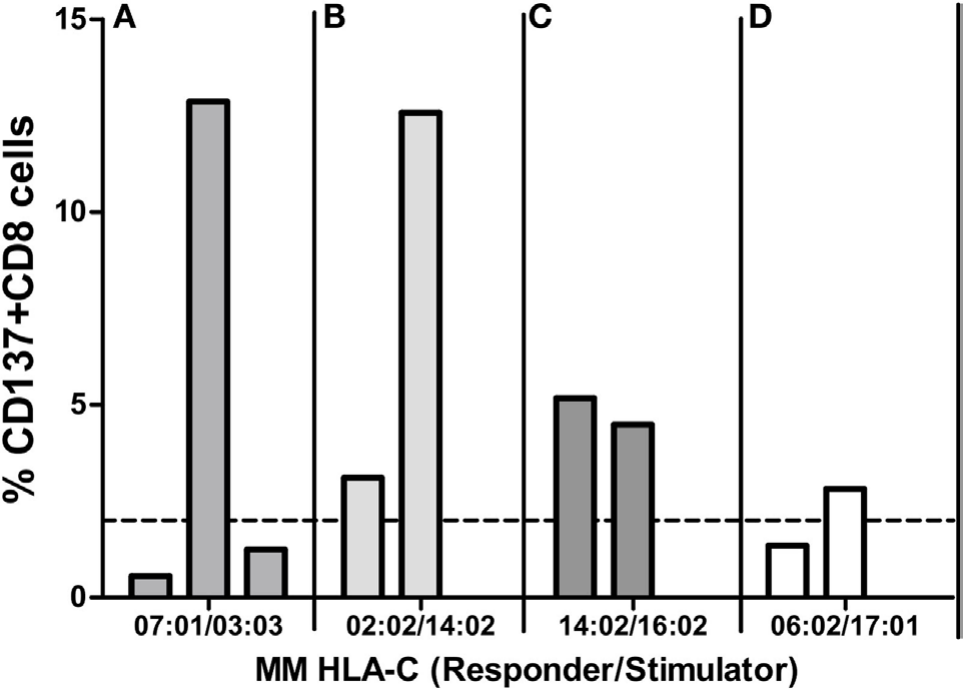
**Mixed lymphocyte reactions (MLRs) between different responders and the same HLA-C MM stimulator**. Each panel represents MLRs between cells of three **(A)** or two **(B–D)** responders and one stimulator. MLRs of each panel were done in parallel (i.e., at the same time) for each of the four experiments **(A–D)** and represent four different HLA-C MMs: the mismatched HLA-C alleles of the responder and the stimulator are indicated below each panel. Alloresponses are given as %ΔCD137^+^CD8^+^ cells (2% cutoff indicated by the dashed line). All pairs tested in **(A–C)** were HLA-DPB1 mismatched. In experiment **(D)**, the two pairs (one positive, one negative) were HLA-DPB1 matched.

We next asked whether CTL allorecognition of the same alloantigen was similar when two different responder cells exhibiting the same two HLA-A~B~C~DRB1~DQB1 haplotypes were tested. As shown in Figure 6A, the C*03:03 MM was recognized by only one of the three C*07:01-positive responders (12.9, 0.56, and 1.2%). In Figure 6B, the C*14:02 MM was much more efficiently recognized by one of the two C*02:02-positive responders (12.6 and 3.1%). On the other hand, allorecognition was more similar in the response to the C*16:02 allele (5.2 and 4.5%) (Figure 6C) and to the C*17:01 allele (1.4 and 2.8%) (Figure 6D). Therefore, the results confirm that the level of HLA-C expression cannot alone account for the strength of the alloresponse because the same incompatibility was recognized quite differently by two (Figure 6B) or three (Figure 6A) responder cells sharing the same HLA-A~B~C~DRB1~DQB1 haplotypes. All pairs tested within the four experiments were HLA-DPB1 incompatible, except the negative pair in the experiment depicted in Figure 6D. This is in accordance with the notion that the TCR repertoire of the responder is a crucial factor that governs allorecognition (27).

### Role of Stimulator Cells

**Figure 7:**
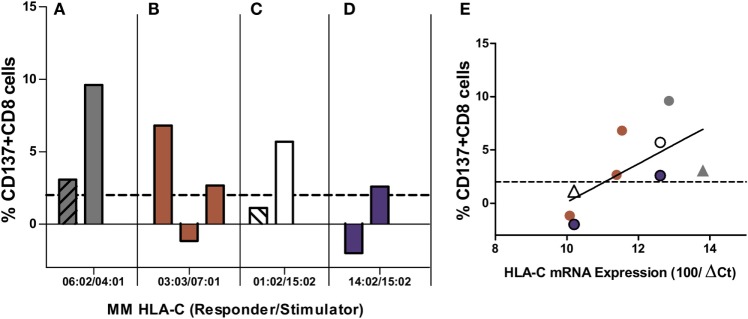
**Mixed lymphocyte reactions (MLRs) between different HLA-C MM stimulators and the same responder**. Each panel represents MLRs between responder cells isolated from the same individual and HLA-C MM stimulator cells from two to three different individuals. MLRs of each panel were performed in parallel (i.e., at the same time) for each experiment and represent four different HLA-C MM: the mismatched HLA-C alleles of the responder and the stimulator are indicated below each panel. Alloresponses are given as %ΔCD137^+^CD8^+^ cells (2% cutoff indicated by the dashed line). All pairs were DPB1 incompatible except two pairs [dashed bars in **(A,C)**]. **(E)** shows the correlation between HLA-C mRNA expression of the stimulator cells and the induced alloresponse (%ΔCD137^+^CD8^+^ cells): *r* = 0.42, *p* = 0.056. Triangles correspond to HLA-DPB1-matched pairs. All other MLRs were HLA-DPB1 mismatched. Colors in **(E)** correspond to those used in **(A–D)**.

In order to avoid the impact of the responders’ TCR repertoire, we compared MLRs of 9/10-matched pairs using a single responder (i.e., 1 single TCR repertoire) and 2–3 stimulator cells from different donors who have exactly the same 2 HLA-A~B~C~DRB1~DQB1 haplotypes with the same HLA-C MM. As shown in four different mismatched combinations (Figure 7), the same MM did not induce the same alloresponse. Only one of the two HLA-C*04:01-positive stimulators was able to induce a high alloresponse of the C*06:02-mismatched responder (3.1 and 9.6%, Figure 7A). C*07:01 expressed in three different stimulator cells was efficiently recognized by the C*03:03 responder in two of the three pairs (6.8, −1.2, and 2.7%, Figure 7B). The C*01:02 responder did recognize the C*15:02 MM in only one of the two stimulator cells (1.1 and 5.7%, Figure 7C). The C*15:02 allele was not or poorly recognized by the C*14:02 responder (−2.9 and 2.3%, Figure 7D). When the levels of HLA-C mRNA were plotted against the %CD137^+^CD8^+^ T cells induced in the alloreponse, the results disclosed a positive but non-significant correlation (*r* = 0.42, *p* = 0.056) (Figure 7E). Furthermore, in two of the four experiments (Figures 7A,C), the stimulator cells that induced the highest alloresponse were HLA-DPB1 mismatched, whereas in the two other experiments, all stimulators were HLA-DPB1 incompatible. Interestingly, among the five positive MLRs with HLA-DPB1 MMs, four of five had a “high expression” phenotype according to Ref. (22).

## Discussion

The success of unrelated HSCT is strongly influenced by HLA matching. For patients with less common HLA haplotypes, 9/10-matched donors with a single HLA-C MM can represent the best option, but such incompatible transplants are characterized by increased risk of posttransplant complications (15, 16, 28). Some clinical studies have proposed that the alloresponse may vary with the nature of the HLA-C MM (12–14), although reliable prediction of less detrimental MMs remains a difficult task. In order to address this question, we measured CTL alloreactivity against incompatible HLA-C alleles in a one-way MLR *in vitro* assay. By quantifiying CD137^+^CD8^+^CD56^−^ T lymphocytes at day 14, we focused on T cell but not NK cell-mediated alloreactivity. The MLR assay had been developed previously to disclose a silent allele MM in the HLA-B44 serotype (23). In parallel, we determined the impact of the expression levels of the mismatched HLA-C alleles as measured by real-time RT-PCR. We also investigated the possible role of mismatched residues in the PBR (α1/α2 domains) that affect peptide and/or TCR binding and the HLA-DPB1-matching status for each pair tested in the MLRs. The outcome of 63 MLR assays showed a large heterogeneity of the alloresponse with 52% positive tests. We first confirmed that MMs differing either outside the α1/α2 domains (C*02:02/02:29, α3 domain) or within the α1/α2 domains but not affecting peptide/TCR binding (C*03:03/03:04, residue 91 in the α2 domain) are not recognized by CD8^+^ alloreactive T cells and could therefore be considered as permissive MMs (28, 29). Next, we confirmed that the C*14:02 MM was able to induce a positive alloresponse in five of seven tested combinations, with two responses among the highest values (>10%) of CD137^+^CD8^+^ T lymphocytes. In addition, the MLRs revealed that C*02:02 or 06:02 MMs were not recognized in 11/12 individuals. Interestingly, a report on CTLp assays performed with HLA-C 9/10-mismatched patient/donor pairs had previously reported that 4/5 C*02:02-positive patients were not recognized by the CTLp assay (30). The overall comparison of the 63 MLRs did not reveal an impact of the HLA-C mRNA expression levels (Figure 2). In particular, a wide distribution of expression levels was observed among the negative MLR combinations. For comparison purposes with previous clinical studies (15, 16), we also analyzed the alloresponses measured in the 63 MLRs on the basis of MFIs taken as proxies of HLA-C expression levels (5, 15). In accordance with the results of the Japan Marrow Donor Program study (16), we did not find any correlation with HLA-C expression using these proxies (Figure 3), although, again, C*14:02 MMs were indeed recognized more efficiently.

Because T cell-mediated alloreactivity is strongly influenced by the peptide repertoire of the stimulating cells (27, 31, 32), we compared the CTL responses with the number of aa residues differences known to impact peptide/TCR binding. As shown in Figure 4, a higher mean number of aa differences was disclosed in the positive MLRs, in accordance with data showing that T cell alloreactivity results from peptide-dependent structural mimicry (27, 32, 33). However, this result was strongly influenced by the C*03:03/03:04 MM pairs and was not confirmed when alloreactivity was considered along a continuous distribution.

An additional genetic factor influencing the alloresponse toward incompatible HLA-C antigens was HLA-DPB1 matching. Indeed, HLA-DPB1 MMs are expected to induce a CD4^+^ T helper response (34) that might increase CTL stimulation as a bystander effect. Clinical studies have demonstrated an impact of DPB1 disparities on the outcome of HSCT (18–20). A higher rate of HLA-DPB1 matching was observed among the negative MLRs (32 versus 14% in the positive pairs). When taking into account both parameters, i.e., ≥10 aa differences in the PBR and HLA-DPB1 MM, a positive correlation with CTL response was disclosed (*p* = 0.021). On the other hand, HLA-DPB1-matching status is not the only important parameter because five MLRs showing wide differences in the ratio of CD137^+^CD8^+^ T cells (Figures 6A,B) were all HLA-DPB1 incompatible.

To avoid variability of alloresponses due to peptide/MHC complexes (polymorphism and expression), we used the same HLA-C MM cells to stimulate different responder cells that were HLA matched among each other. Since in three of four experiments, a given HLA-C MM was able to induce different alloresponses when tested against two or three different responders with exactly the same HLA haplotypes (Figure 6), this indicates that CTL activation is modulated by the TCR repertoire of the responder cells. HLA-C-restricted T lymphocytes are important actors of antiviral immunity [reviewed by Blais et al. (35)]. Because HLA-C is less saturated with endogenous peptides (36), access to viral peptides might increase the repertoire diversity of peptides bound to mismatched HLA-C antigens and thereby increase the relative frequencies of cross-reactive CTLs. TCR repertoire analysis of the responding cells should help in better understanding the variability of the T cell alloresponse.

Nevertheless, when both the effects of the number of aa MMs and of TCR repertoire were neutralized by stimulating the same responder cells expressing the same HLA-C MM on identical HLA haplotypes, variable alloresponses were observed (Figures 7A–D). In this case, CTL activation did show some weak correlation with HLA-C mRNA expression of the stimulator allele (Figure 7E), although this result should be interpreted cautiously because in two of the four experiments the low (Figure 7A) or negative (Figure 7C) alloresponse corresponded to the HLA-DPB1-matched pairs.

We acknowledge some limitations of our study. The *in vitro* MLR assay can only be a simplified model of the alloreaction occurring in HSCT. Yet, it allowed to confirm the permissiveness of the C*03:03/03:04 MM as well as the higher risk of aGVHD and transplant-related mortality conferred by patient’s C*14:02 MM, which were indeed reported by clinical studies (15, 16, 29). We are aware that HLA-C mRNA steady-state amounts were determined in PBMC and therefore may not allow disclosing variability in HLA-C expression in different tissues and cell types (3), which could be relevant to GVHD. Finally, this study focused on T cell alloreactivity and did not address NK cell-mediated responses that are HLA-C alloantigen-dependent through interaction with killer-immunoglobulin-like receptors and have been shown to be particularly relevant in haploidentical HSCT (37, 38).

Taken together and carefully controlling for potential confounding variables, our results suggest that HLA-C allorecognition in mismatched HSCT depends on the number of aa MM residues in the PBR, on HLA-DPB1 matching, on the TCR repertoire of the responding cells (although not measured directly in our experiments), and possibly on HLA-C expression. However, the complex interplay of these genetic factors is not straightforward. Clearly, neither HLA-C mRNA expression levels nor cell surface expression measured on heterozygous individuals could reliably predict the strength of the alloresponse, as determined by an *in vitro* assay. Therefore, when selecting a partially matched unrelated donor for HSCT, we recommend that HLA-C MMs with ≥10 aa MM in the PBR and with a concomitant HLA-DPB1 MM should be avoided. However, in specific cases, HLA-DPB1 mismatching could be beneficial for the graft-versus-leukemia effect (39, 40). The identification of C*06:02 as a potentially permissive MM supports the importance of HLA-DPB1 matching because five of the six negative pairs were HLA-DPB1 matched, although they were characterized by >10 aa MMs in the PBR and by high levels of expression. Most likely, these results might be extrapolated to HLA-A or -B MMs, but this remains to be investigated in retrospective clinical studies or by *in vitro* assays. Thus for unrelated HSC donor searches that allow to procure only donors with single HLA class I disparities, this study confirms that it should be beneficial to prioritize HLA-DPB1-compatible donors. It also emphasizes that a pretransplant *in vitro* MLR assay with patient and donor lymphocytes, such as the one developed in this study, is a reliable way to predict CTL alloreactivity.

## Author Contributions

FB and J-MT conceived and designed the experiments. FB performed the experiments. SB performed the statistical analyses. FB and SB designed the figures. J-MT wrote the first draft of the manuscript, and FB and SB contributed to the writing of the manuscript.

## Conflict of Interest Statement

The authors declare that the research was conducted in the absence of any commercial or financial relationships that could be construed as a potential conflict of interest.

## Abbreviations

HSCT, hematopoietic stem cell transplantation; aa, amino acid; MLR, mixed lymphocyte reaction; TCR, T cell receptor; PBR, peptide-binding region; MM, mismatch.
